# *Cupriavidus necator*-Produced Polyhydroxybutyrate/Eudragit FS Hybrid Nanoparticles Mitigates Ulcerative Colitis via Colon-Targeted Delivery of Cyclosporine A

**DOI:** 10.3390/pharmaceutics14122811

**Published:** 2022-12-15

**Authors:** Juho Lee, Aruzhan Saparbayeva, Shwe Phyu Hlaing, Dongmin Kwak, Hyunwoo Kim, Jihyun Kim, Eun Hee Lee, Jin-Wook Yoo

**Affiliations:** 1 College of Pharmacy, Pusan National University, Busan 46241, Republic of Korea; 2 College of Pharmacy, Korea University, Sejong 30019, Republic of Korea

**Keywords:** polyhydroxybutyrate, pH-responsive, ulcerative colitis, colitis-targeted drug delivery, sustained release, PHB biosynthesis, *Cupriavidus necator*

## Abstract

Polyhydroxybutyrate (PHB) has emerged as a novel material for replacing various plastics used in the medical field. However, its application as a drug-delivery carrier for colitis-targeted delivery has not been explored. In this study, we used biosynthesized PHB combined with Eudragit FS (EFS) and cyclosporine A (CSA) to develop pH-responsive controlled CSA-releasing nanoparticles (CSA-PENPs) for colitis-targeted drug delivery and demonstrated its enhanced therapeutic efficacy in a dextran sulfate sodium (DSS)-induced murine colitis model. PHB was successfully biosynthesized in the bacterium *Cupriavidus necator*, as demonstrated by ^1^H-NMR and FT-IR analyses. CSA-PENPs were fabricated via the oil-in-water emulsion solvent evaporation method. Owing to the potent pH-responsive and sustained drug release properties provided by PHB and EFS, CSA-PENPs could deliver a sufficient amount of CSA to inflamed tissues in the distal colon; in contrast, CSA-loaded EFS nanoparticles displayed premature burst release before reaching the target site. Due to enhanced CSA delivery to colitis tissues, CSA-PENPs exhibited potent anti-inflammatory effects in the DSS-induced murine colitis model. Overall, CSA-PENPs could be a promising drug-delivery system for treating ulcerative colitis.

## 1. Introduction

Polyhydroxybutyrate (PHB) is a naturally derived biocompatible and biodegradable polyester synthesized by various microorganisms as a carbon energy-storage material [1,2]. To date, various physicochemical and biological characteristics of PHB, an alternative to petroleum-based polymers, have been elucidated, and its potential for biomedical use has been highlighted owing to its desirable properties, such as biocompatibility and biodegradability [3]. As PHB and its degradation product (hydroxybutyric acid) are naturally occurring materials in the human body, PHB is recognized as a highly biocompatible polymer [4]. In addition to its biocompatibility, PHB has physical properties similar to those of polyethylene (PE) and polypropylene (PP), which are the most widely used petroleum-based versatile polymers [2,5]. As a result, PHB has attracted considerable attention as a promising material for medical use. In particular, the product was approved by the FDA for surgical applications in 2007 [6,7].

In addition to the development of PHB-based products for medical use, applications of PHB for drug delivery have been explored owing to its biocompatibility and the sustained release property provided by its appropriate water-barrier properties [8]. In particular, the potent sustained release effect would be a desirable property for colon-targeted drug-delivery systems that must protect and prevent the release of the incorporated drugs in the stomach and small intestine. Therefore, PHB could be a good colon drug-delivery carrier for treating colon-specific diseases, including ulcerative colitis (UC).

UC is a chronic inflammatory disease that originates in the colorectum due to various factors, including the dysbiosis of microbiota, genetic susceptibility, immune responses, and environmental factors [9,10,11]. Owing to severe inflammation in the distal colon, patients with UC suffer from several symptoms, such as rectal bleeding, diarrhea, and abdominal pain [12,13]. Moreover, patients with UC are at an increased risk for developing colorectal cancer, which has the second highest mortality rate worldwide [14,15,16]. To date, the exact pathogenesis of UC has not been elucidated, and a cure for UC has not been developed [17,18]. Therefore, current strategies for treating UC aim to alleviate inflammation and prevent GI tract disruption [19,20]. As a result, various anti-inflammatory therapeutics, such as corticosteroids and immunosuppressants, are available in the clinic to treat UC [21,22]. However, owing to a lack of targeted delivery systems, current anti-inflammatory therapies using these drugs can cause severe side effects, such as renal toxicity, lymphoma, hypertension, seizures, and opportunistic infections [23,24,25]. In addition, only few drug molecules can accumulate in inflamed tissues in the colon, resulting in diminished anti-inflammatory effects [26]. To overcome these limitations, colon-targeted oral drug-delivery systems are being assessed, as they can reduce systemic side effects via inhibition of the drug release and absorption in the stomach and small intestine, and increase drug exposure in inflamed tissues in the colon [27].

As colon-targeted drug-delivery systems, pH-responsive formulations are one of the most widely used. Furthermore, marketed products, such as Asacol^®^, Ipocol^®^, Lialda^®^, and Mesavant^®^, are available in the clinic owing to their potent enteric protective effects in the stomach and small intestine [26,28]. In particular, pH-responsive nanoparticles have emerged as alternatives, as they can effectively accumulate in damaged colon tissues [29]. However, pH-responsive nanoparticles exhibit premature burst drug release in the distal ileum and proximal colon, resulting in unwanted systemic absorption and a lack of drug accumulation in colitis tissues located in the distal parts of the colon [30,31,32]. Thus, additional sustained drug release properties should be evaluated for the development of a desirable colitis-targeted drug-delivery system.

In this study, we hypothesized that the addition of PHB to pH-responsive nanoparticles would overcome these limitations as the potent sustained drug release property of PHB, by preventing water invasion into the nanoparticles, could prevent premature burst release in the distal ileum and proximal colon. Moreover, continuous drug release during colon passage could provide more drugs to inflamed colon tissues, resulting in enhanced anti-inflammatory effects. Herein, to develop a PHB-based pH-responsive controlled releasing nanoparticle for UC treatment, we designed cyclosporine A (CSA)-loaded PHB/Eudragit FS (EFS) nanoparticles (CSA-PENPs). CSA is a widely used immunosuppressant due to its potent anti-inflammatory effects; however, without a targeted delivery system, severe systemic toxicity is unavoidable. Further, decreased drug concentration in the inflamed tissue limits the therapeutic efficacy of CSA [26]. Therefore, CSA is a good model drug for developing colitis-targeted drug-delivery systems. The pH-responsive EFS moiety may protect CSA release in the stomach and small intestine, and the PHB moiety could prevent the premature burst release of CSA in the proximal colon. After reaching the distal colon, CSA-PENPs could release CSA in a sustained manner resulting in enhanced CSA accumulation in damaged colitis tissues. After the biosynthesis and purification of PHB, CSA-PENPs were fabricated, and their physicochemical properties were examined via a series of in vitro experiments. The in vivo distribution and anti-inflammatory effects of CSA-PENPs were also evaluated in a dextran sulfate sodium (DSS)-induced murine colitis model.

## 2. Materials and Methods

### 2.1. Materials

CSA and polyvinyl alcohol (PVA) were purchased from Sigma-Aldrich (St. Louis, MO, USA). The EFS was generously provided by Evonik Korea, Ltd. (Seoul, Korea). Bacto tryptic soy broth (TSB) was purchased from BD Biosciences (San Jose, CA, USA). Mayer’s hematoxylin solution was purchased from Wako Pure Chemical Industries (Osaka, Japan). DSS (molecular weight: 40 kDa) and 11-Chloro-1,1′-di-n-propyl-3,3,3′,3′-tetramethyl-10,12-trimethyleneindatricarbocyanine iodide (IR780) were purchased from Alfa Aesar (Haverhill, MA, USA). The Eosin Y solution was purchased from Daejung Chemicals and Metals (Shiheung, Republic of Korea). All other chemicals, reagents, and solvents were of the highest commercially available analytical grade.

### 2.2. Preparation of PHB

#### 2.2.1. Microorganism

*C. necator* (KCTC No. 22469, ATCC No. 17699) was purchased from the Korean Collection for Type Cultures (KCTC, Daejon, Republic of Korea). *C. necator* was cultured in TSB medium at 30 °C for 24 h and stored at −80 °C after cryoprotection with 30% glycerol for future use.

#### 2.2.2. PHB Biosynthesis, Extraction, and Purification

PHB was prepared from *C. necator* according to a previously reported method, with some modifications [33,34,35]. Briefly, 1 mL of *C. necator* suspension stored at −80 °C was precultured in 50 mL of TSB media for 24 h at 30 °C. After preculture, the suspension was transferred to 2 L of fresh TSB medium and cultured for 48 h at 30 °C. For PHB biosynthesis, *C. necator* was collected via centrifugation (2000× *g*, 5 min) and was added to 2 L of modified mineral salts media (MMM); 1 L of MMM contained 20 g of fructose, 1.5 g of ammonium sulphate, 4.6 g of potassium dihydrogen phosphate, 4.1 g of sodium monobasic phosphate, 0.5 g of magnesium sulphate heptahydrate, and 2 mL of trace element solution; pH adjusted to 7. The trace element solution contained 2 g of iron(II) sulfate heptahydrate, 2 g of calcium chloride dihydrate, 0.3 g of boric acid, 0.2 g of cobalt(II) chloride hexahydrate, 0.1 g of zinc sulfate heptahydrate, 0.03 g of manganese(II) chloride tetrahydrate, 0.03 g of sodium molybdate dihydrate, 0.02 g of nickel(II) chloride hexahydrate, and 0.01 g of copper(II) chloride dihydrate in 1 L of 0.1 N HCl solution. After an additional incubation for 72 h at 30 °C, the PHB-containing *C. necator* biomass was collected via centrifugation (2000× *g*, 5 min) and lyophilized for future use.

Before PHB extraction, 50 mL acetone was added to 5 g PHB-containing *C. necator* biomass and incubated for 24 h at room temperature. After filtration and drying, the PHB was extracted from the biomass via reflux extraction using 200 mL chloroform for 72 h at 60 °C. The supernatant was collected via filtration, and 1 L of EtOH was added to precipitate the PHB from the solution. The PHB was collected and vacuum dried for future use.

To characterize PHB from *C. necator*, ^1^H nuclear magnetic resonance (NMR) spectroscopy was conducted after dissolving PHB in CDCl_3_ at 500 MHz (Varian Unity Inova 500 spectrometer, Palo Alto, CA, USA). Fourier-transform infrared (FT-IR) spectra of the biosynthesized PHB were obtained via the KBr method using a Nicolet iS50 spectrometer (Thermo Fisher Scientific, Waltham, MA, USA).

### 2.3. Preparation of CSA-PENPs

CSA-PENPs were fabricated using the oil-in-water (O/W) emulsion solvent evaporation method. Briefly, PHB (25 mg), EFS (25 mg), and CSA (5 mg) were dissolved in chloroform (5 mL). The solution was then introduced into 25 mL of a 1% PVA solution and sonicated at 150 W for 2.5 min. Following the addition of 10 mL of distilled water (DW), the emulsion was stirred at 550 rpm for 4 h at room temperature to remove chloroform. After solvent removal, the CSA-PENPs were collected via centrifugation (20,000× *g*, 20 min) and washed twice with DW. CSA-PENPs were cryoprotected by adding 25 mg trehalose and stored at −20 °C after freeze-drying. For in vivo imaging, IR780-loaded CSA-PENPs and ENPs were fabricated using the above method, except for the addition of 0.5 mg of IR780 in the chloroform solution-preparation step.

To characterize the CSA-PENPs, particle morphology, size, zeta potential, and CSA loading were analyzed. The morphology of the nanoparticles was examined using scanning electron microscopy (SEM, Supra 25, Carl Zeiss, Jena, Germany). After dispersing in DW (pH adjusted to 7.4), the mean hydrodynamic particle size and zeta potential of the CSA-PENPs were measured using a Zetasizer Nano ZS90 (Malvern Instruments, Worcestershire, UK). To calculate the CSA loading and encapsulation efficiency, the CSA formulations were dissolved in acetonitrile and sonicated at 30 °C for 30 min to extract CSA. Thereafter, the samples were centrifuged for 10 min at 17,000× *g*. The supernatant was collected, and the CSA concentration was measured using high-performance liquid chromatography (HPLC, VDSpher 100 C18-E column (4.6 mm × 150 mm, 3.5 μm, VDS Optilab, Berlin, Germany), mobile phase: acetonitrile/H_2_O (75/25), flow rate: 1.5 mL/min, oven temperature: 65 °C). The signals were measured using an ultraviolet detector (SPD-20A; Shimadzu, Kyoto, Japan) at a wavelength of 210 nm. A calibration curve using CSA was obtained over the range of 2–2000 μg/mL.

### 2.4. Drug Release Study

The CSA release profiles of the formulations were analyzed in gradually changing pH media (pH 1.2, 6.8, and 7.4) to simulate the pH conditions of the stomach, small intestine, and large intestine, respectively. 0.2% of tween 80 was added to the releasing media to solubilize the released CSA. the CSA formulations were dispersed in a dialysis bag (Sigma Aldrich, 14,000 MWCO) and incubated in media (pH 1.2) for 0 to 2 h at 37 °C. Two hours after the initiation of the experiment, the pH was changed from 1.2 to 6.8, and the mixture was incubated for 3 h (time point of 2 h to 5 h) at 37 °C. At 5 h, the pH was changed from 6.8 to 7.4, and the mixture was incubated for up to 24 h at 37 °C. At set time points, the supernatant was harvested, and the CSA concentration was measured via HPLC following the method mentioned above. The percentage of CSA that could be released under the simulated condition of the colorectum was calculated by measuring the drug release from 7 to 24 h.

### 2.5. Development of the DSS-Induced Murine Colitis Model

All animal experiments were reviewed and approved by the Pusan National University Institutional Animal Care and Use Committee (PNU-IACUC) on 16 March 2022 (PNU-2022-0089). ICR mice (6 weeks old, male) were purchased from Samtako Bio Korea (Osan, Republic of Korea) and acclimatized for 1 week before initiation of the experiment. For colitis induction, 3% of DSS in DW was administered in drinking water for seven days.

### 2.6. In Vivo Distribution of CSA-PENPs

The in vivo distribution of the CSA formulations after oral administration was evaluated using an in vivo imaging system (FOBI; Neoscience, Suwon, Republic of Korea), following the previously reported method with some modifications [36]. Briefly, after 24 h of fasting, 5 mg of IR780-loaded CSA-PENPs and ENPs were administered orally. All mice were euthanized 15 h after administration and their GI tract (stomach to anus) was excised for in vivo imaging. The fluorescence signal from the GI tract was measured and calculated using the Neoimage FOBI software (Neoscience).

### 2.7. Evaluation of In Vivo Therapeutic Efficacy

#### 2.7.1. Macroscopic Assessments

The therapeutic effects of the CSA formulations were evaluated using a DSS-induced colitis model following a previously reported method [20]. After colitis induction (on day 7), mice were randomly divided into four groups: CSA solution, CSA-ENPs, CSA-PENPs, and untreated colitis groups. Healthy mice were used as controls. The CSA formulations were administered orally (15 mg/kg of CSA equivalent daily) for six days. To evaluate colitis severity, body weight loss, stool consistency, and rectal bleeding were monitored as clinical parameters, and the disease activity index (DAI) was calculated following a previously reported method [37,38]. Briefly, body weight loss was measured daily and scored according to the following criteria: <0% (0 point), 0–0.9% (1 point), 1–4.9% (2 points), 5–9.9% (3 points), 10–19.99% (4 points), and >20% (5 points). Stool consistency was observed and scored as follows: well-formed stools (0 points), only detectable pasty stools in the anus (1 point), distinct pasty stools localized in the anus (2 points), pasty stools slightly covering the anus (3 points), pasty stools distinctly covering the anus (4 points), and massive pasty stools covering the anus (5 points). Rectal bleeding was scored as follows: no bleeding (0 points), detectable bloodstain in the anus (1 point), slight bleeding localized in the anus (2 points), bleeding localized in the anus (3 points), distinct bleeding and presence of bloodstain around the anus (4 points), and gross bleeding and bloodstain around the anus (5 points). DAI was calculated by summing the scores of body weight loss, stool consistency, and rectal bleeding.

At the last day of the experiment, all mice were euthanized, and the colon and spleen were excised for macroscopic evaluation of colitis severity. After imaging of the excised colon and spleen, colon length and spleen weight were measured as indicators of colitis.

#### 2.7.2. Histological Assessments

For histological examination, hematoxylin and eosin (H&E) staining was conducted following a previously reported method, with some modifications [39,40]. Briefly, each colon sample was collected and processed into a Swiss-rolled sample [41]. After fixing in 10% formalin solution for 24 h, the samples were embedded in paraffin blocks and sliced into 5 μm-thick sections. After sectioning, H&E staining was performed following the manufacturer’s protocol. The histological images were obtained using a light microscope (BX53; Olympus, Tokyo, Japan) at a magnificence of 10×. Histological scores were calculated from the H&E images following a previously established method, with some modifications [42,43]. Briefly, each H&E image was scored according to the following criteria: lack of change (0 points), slight superficial mucosal damage (1 point), the presence of pathological lesions (2 points), the presence of distinct and more diffuse lesions (3 points), the presence of intensive mucosal damage (4 points), and totally disrupted tissues (5 points). The histological score was calculated by summing the scores from three different portions of the colon in each experimental group. Grading was performed blindly on coded slides and triplicated.

### 2.8. Statistical Analysis

Data are presented as mean ± standard deviation (SD), and all experiments were conducted at least three times. All statistical analyses were performed using GraphPad Prism 5.0 (GraphPad Software, La Jolla, CA, USA). Statistical analyses were performed using a two-tailed t-test, one-way analysis of variance (ANOVA), or two-way ANOVA followed by the Bonferroni post hoc test or Tukey’s post hoc test. A *p*-value less than 0.05 was considered to indicate a statistically significant difference.

## 3. Results and Discussion

### 3.1. Preparation of PHB

**Figure 1:**
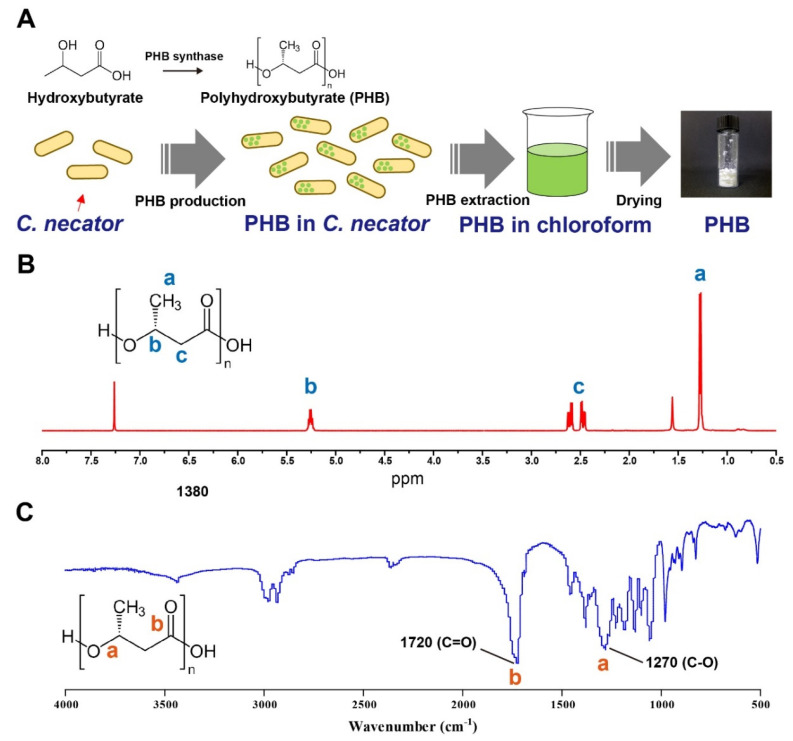
Preparation of polyhydroxybutyrate (PHB). (**A**) Schematic diagram of PHB preparation. (**B**) 1H-NMR spectra of PHB dissolved in CDCl3 solution. (**C**) FT-IR spectra of the PHB.

PHB was prepared using three sequential processes: bacterial growth and PHB biosynthesis in *C. necator,* purification using acetone, and extraction with chloroform (Figure 1A). For PHB biosynthesis, *C. necator* was selected owing to its various advantages as an efficient PHB-producing bacterium; these advantages include robust growth conditions (facultative anaerobe) and the remarkable capacity for PHB production and accumulation [44]. After PHB biosynthesis, PHB-containing *C. necator* was collected and freeze-dried. The biomass was incubated in acetone to remove impurities, including lipids. After purification, the PHB in the biomass was extracted using hot chloroform for 3 days. During the extraction step, PHB was solubilized in chloroform, resulting in a color change (clear to light yellowish) and slightly increased viscosity. After collecting the supernatant, PHB was precipitated using ethanol. Owing to the poor solubility of PHB in ethanol, pure PHB was obtained by collecting the precipitant. To characterize PHB, ^1^H-NMR spectroscopy was conducted, and the characteristic peaks of PHB were examined via a comparison with those of previously reported PHB. As shown in Figure 1B, characteristic peaks were clearly observed at 1.2, 2.5, and 5.2 ppm, which corresponded to -CH_3_, -CH_2_, and -CH in the PHB molecule, respectively. The results also aligned with previously reported characteristic peaks of PHB [45,46,47]. For further examination, the FT-IR spectra of PHB were measured. As shown in Figure 1C, distinct characteristic peaks stretching from C=O and C-O were clearly observed. The characteristic peak stretch of the biosynthesized PHB had high similarity to that of previously reported PHB [48,49], indicating that PHB was successfully biosynthesized and extracted using the optimized method.

### 3.2. Fabrication of CSA-PENPs

**Figure 2:**
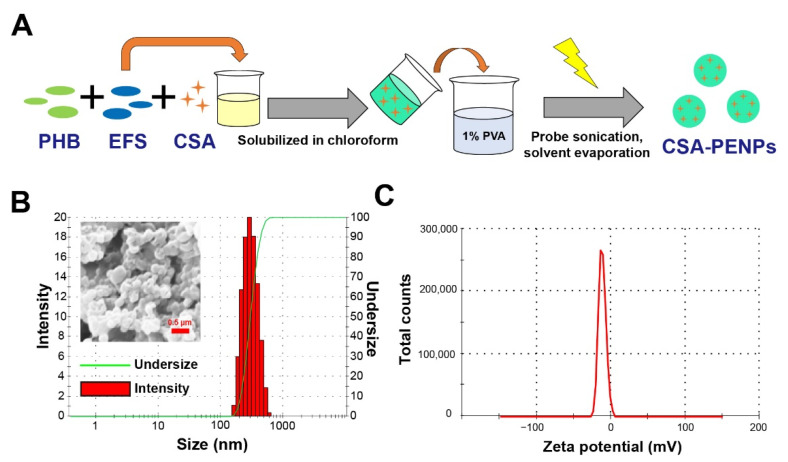
Fabrication of CSA-PENPs. (**A**) Schematic diagram for CSA-PENPs fabrication. (**B**) Hydrodynamic particle size distribution and representative SEM image of CSA-PENPs. (**C**) Zeta potential distribution of CSA-PENPs in the pH 7.4 medium.

CSA-PENPs were successfully fabricated using the oil-in-water emulsion-solvent evaporation method (Figure 2A). As shown in Figure 2B, global-shaped nanoparticles were observed in the SEM images, with a mean particle diameter of 311.4 ± 9.1 nm and polydisperse index (PDI) of 0.136 ± 0.010. The zeta potential of the CSA-PENPs was −18.4 ± 0.06 mV, which indicates that the surface of the CSA-PENPs was slightly negatively charged (Figure 2C). As submicron-sized particles can easily accumulate in the inflamed area [15,50] and the slightly negatively charged nanoparticles could exhibit enhanced mucus penetration effects compared to the positively charged nanoparticles [51,52], CSA-PENPs could deliver CSA to the inflamed tissues via enhanced mucus penetration and inflamed tissue accumulation. To determine the CSA content in CSA-PENPs, the amount of CSA was measured using HPLC. As shown in Table 1, CSA was successfully loaded into the CSA-PENPs with a CSA loading of 6.28% and encapsulation efficacy of 69.1%. As CSA possesses poor water solubility [53], drug loss during nanoparticle fabrication is minimized, resulting in adequate CSA loading and encapsulation efficacy as a colitis-targeted nanoparticle drug-delivery system.

### 3.3. Drug Release Study

**Figure 3:**
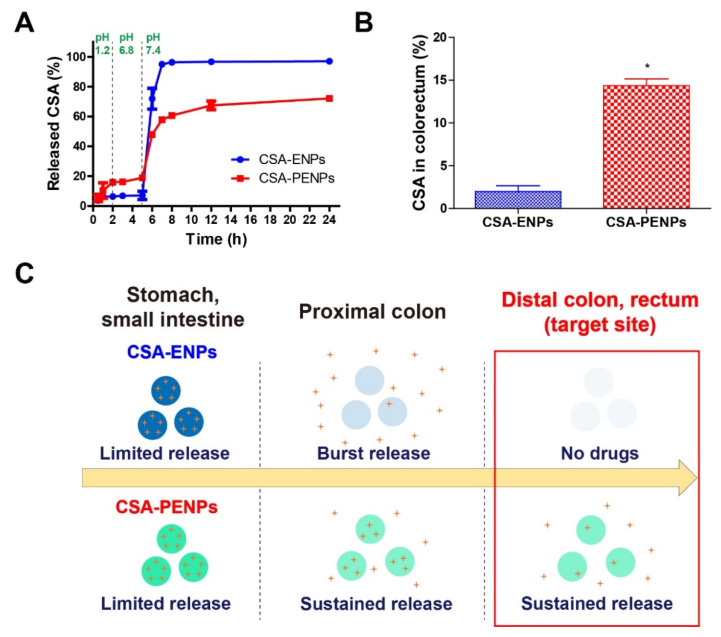
Drug release profiles of the CSA formulations in the gradually pH-changing media. (**A**) Drug release profiles of CSA-ENPs and CSA-PENPs. (**B**) Percentage of the released CSA in the simulated conditions of the colorectum. (**C**) Suggested mechanisms for enhanced oral CSA delivery to the distal colon. The results are expressed as mean ± standard deviation (n = 3, * *p* < 0.05 compared to the CSA-ENPs group).

The drug release profile of CSA-PENPs was examined by gradually changing the pH of the media. The pH of the releasing medium was gradually increased to simulate GI tract passage (stomach, small intestine, and large intestine with pH values of 1.2, 6.8, and 7.4, respectively). As shown in Figure 3A, for 0–5 h, under the simulated pH conditions of the stomach and small intestine (pH 1.2, 6.8), both CSA-ENPs and CSA-PENPs showed limited CSA release (less than 20% of the CSA was released). In particular, due to the poor solubility of EFS at pH < 7, only 7% of the CSA was released from the CSA-ENPs under the pH 1.2 and 6.8 conditions. After exposure to pH 7.4, the CSA-ENPs exhibited a burst release of CSA (more than 80% of CSA was released for 2 h) owing to the rapid dissolution of the EFS layer. In contrast, CSA-PENPs released approximately 38% of CSA for 2 h after being exposed to the pH 7.4 media, and the other remaining drugs in the nanoparticles were released for more than 24 h in a sustained manner. To evaluate the available CSA at the target site (distal colon), the CSA percentage released during the time points of 7 to 24 h was calculated from the CSA release profiles (Figure 3B). As more than 80% of the CSA in CSA-ENPs was prematurely released, less than 2% of CSA could be released around the target site. On the other hand, a 7-fold higher amount of CSA (14%) was released from the CSA-PENPs in the simulated conditions of the distal colon within 24 h. As UC lesions are commonly located in the distal colon, immediate release of CSA after exposure to pH 7.4 could cause drug loss before reaching the distal colon. Thus, as shown in Figure 3C, owing to the burst CSA release from the CSA-ENPs, most of the CSA molecules were released in the proximal colon and only few molecules accumulated in the inflamed tissues. In contrast, following the initial burst release of CSA after exposure to media at pH 7.4, long-term exposure of CSA to the inflamed tissues in the distal part of the colon could be achieved via oral administration of CSA-PENPs. The sustained CSA release from CSA-PENPs may have been due to the delayed dissolution effect of the EFS from the PHB-EFS hybrid layer provided by the insoluble PHB moiety in pH 7.4 media.

### 3.4. In Vivo Distribution Examination

**Figure 4:**
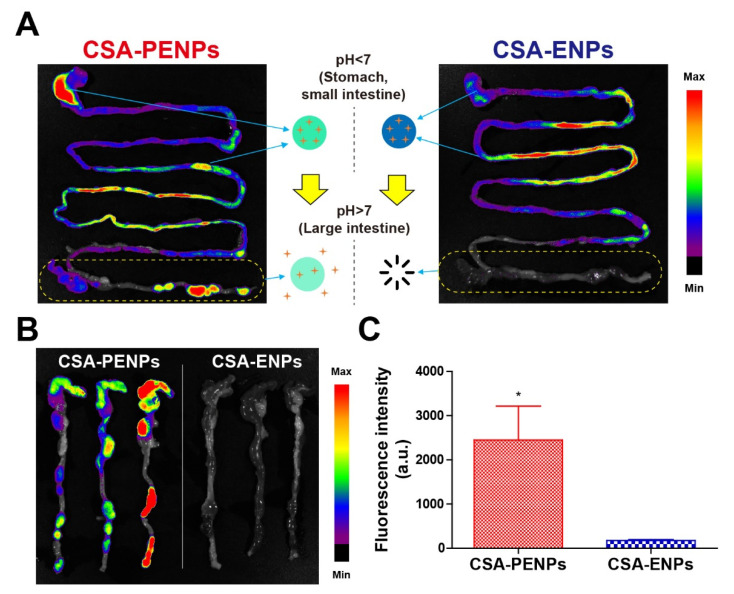
In vivo distribution of the CSA formulations at 15 h after oral administration. (**A**) Fluorescence images of the whole gastrointestinal tract. Yellow dotted rectangle indicates the large intestine. (**B**) Fluorescence images of the large intestine. (**C**) Fluorescence intensity of the large intestine. Data are expressed as mean ± standard deviation (n = 3, * *p* < 0.05 compared to the CSA-ENPs group).

To assess the in vivo distribution of the CSA formulations after oral administration, IR780-loaded CSA-ENPs and PENPs were used instead of CSA-ENPs and PENPs. Images were obtained 15 h after oral administration, which is a sufficient time point for the nanoparticles to reach the large intestine. Fluorescence signals were clearly observed in the colorectum of the CSA-PENP-treated group, whereas signals were not observed in the CSA-ENP-treated group (Figure 4A,B). For quantitative analysis, the fluorescence intensity of the IVIS images of the large intestine was measured. As shown in Figure 4C, the CSA-PENP-treated group had a significantly higher fluorescence signal than the CSA-ENP-treated group (*p* < 0.05). Owing to the rapid dissolution of the CSA-ENPs at pH values higher than 7, the nanoparticles could not reach the colon where the UC lesions existed. Despite the remarkable enteric protective effects, owing to the premature burst drug release at the end of the small intestine and proximal colon, CSA-ENPs could not accumulate in the inflamed tissues located in the distal colon, resulting in insufficient CSA concentration at the target site. In contrast, owing to sustained drug release in the colonic environment, distinct fluorescence signals from CSA-PENPs were detected in the colorectum. The results suggest that CSA-PNEPs could reach the colorectum and provide sufficient CSA to the inflamed tissues, resulting in enhanced anti-inflammatory effects for UC treatment.

### 3.5. In Vivo Therapeutic Efficacy of CSA-PENPs

#### 3.5.1. Macroscopic Analysis of Colitis

**Figure 5:**
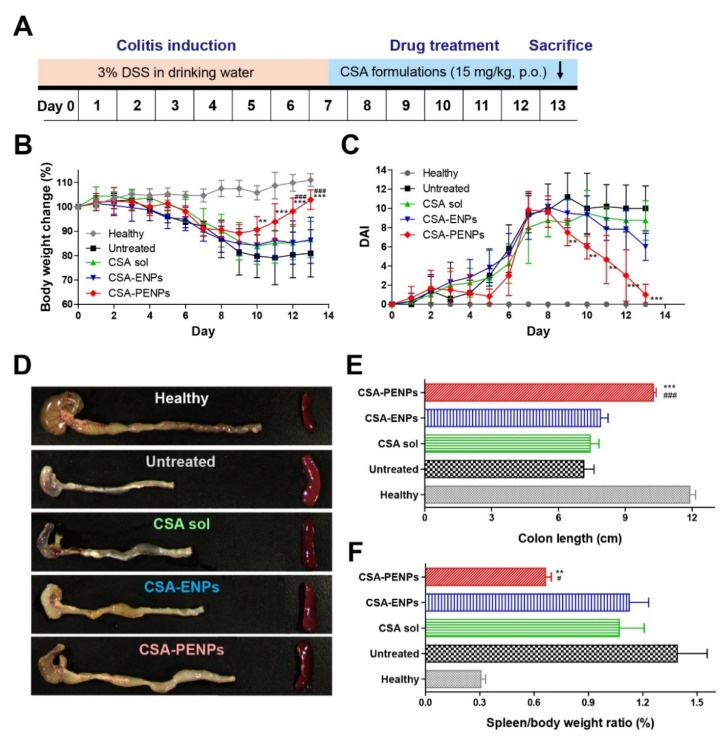
In vivo therapeutic efficacy of CSA-PENPs in the DSS-induced colitis model. (**A**) Experimental flow. (**B**) Body weight change profiles. (**C**) Change profiles for the DAI scores. (**D**) Representative macroscopic images of the excised large intestine on day 13. (**E**) Colon length on day 13. (**F**) Spleen per body weight ratio on day 13. ** and *** denote *p* < 0.01 and *p* < 0.001, respectively, compared to the untreated colitis group. ^#^, and ^###^ denote *p* < 0.05, and *p* < 0.001, respectively, compared to CSA-ENP-treated group. Data are expressed as mean ± standard deviation (n ≥ 4).

The in vivo therapeutic efficacy of CSA-PENPs was determined using a DSS-induced colitis mouse model. After 7 days of 3% DSS administration to induce UC, the CSA formulations (15 mg/kg CSA) were orally administered daily for 6 days (Figure 5A). During this period, the body weight and DAI were recorded to evaluate the severity of colitis (Figure 5B,C). Owing to DSS administration, the body weight decreased in all groups up to day 7. After CSA-PENP treatment initiation, body weight reduction was stopped, while the body weight of the other groups continued to decrease. The body weight of the CSA-PENP-treated group started to recover from two days after treatment initiation and was significantly higher than that of the untreated colitis group (*p* < 0.01). In addition to the body weight profiles, the change in the DAI score of the CSA-PENP-treated group was significantly different from that of the other groups. As shown in Figure 5C, the DAI of all groups increased until day 7 after DSS administration. Three days after treatment initiation, the DAI scores of the CSA-PENP-treated group were significantly lower than those of the untreated colitis group (*p* < 0.01). As CSA-PENPs could accumulate in the inflamed tissues and provide sufficient CSA, inflammatory signs, such as body weight reduction and increasing DAI scores, were effectively ameliorated by CSA-PENP treatment. In contrast, the CSA solution and CSA-PENP-treated group did not display significant therapeutic effects compared to the untreated colitis group due to the failure in colitis-targeted delivery, resulting in an insufficient CSA level at the target site.

Colon length and spleen/body weight ratio were measured as parameters of colitis severity on day 13. Owing to severe inflammation, the colon length of the untreated group was shorter than that of healthy mice (Figure 5D,E). Treatment with the CSA solution or CSA-ENPs did not affect colon length, indicating that these treatments could not mitigate inflammatory reactions in the colon. However, after 6 days of treatment with CSA-PENPs, the colon length was significantly recovered compared to that of the CSA-ENP-treated and untreated group (*p* < 0.001), and displayed the potent anti-inflammatory effects provided by the targeted delivery of CSA to the colitis tissues. The enhanced anti-inflammatory effects of CSA-PENPs were also confirmed by the spleen/body weight ratio assessment. As shown in Figure 5F, the spleen/body weight ratio was markedly increased in the CSA solution, CSA-ENP-treated and untreated groups resulted from the severe inflammation. On the other hand, that of the CSA-PENP-treated group was significantly reduced compared to that of the untreated and CSA-ENP-treated groups (*p* < 0.001 and *p* < 0.05, respectively) due to the potent anti-inflammatory effects of the CSA-PENPs. Taken together, owing to the enhanced CSA accumulation in inflamed lesions in the distal colon via colitis-targeted delivery, CSA-PENPs exhibited potent anti-inflammatory effects.

#### 3.5.2. Histological Analysis of Colitis

**Figure 6:**
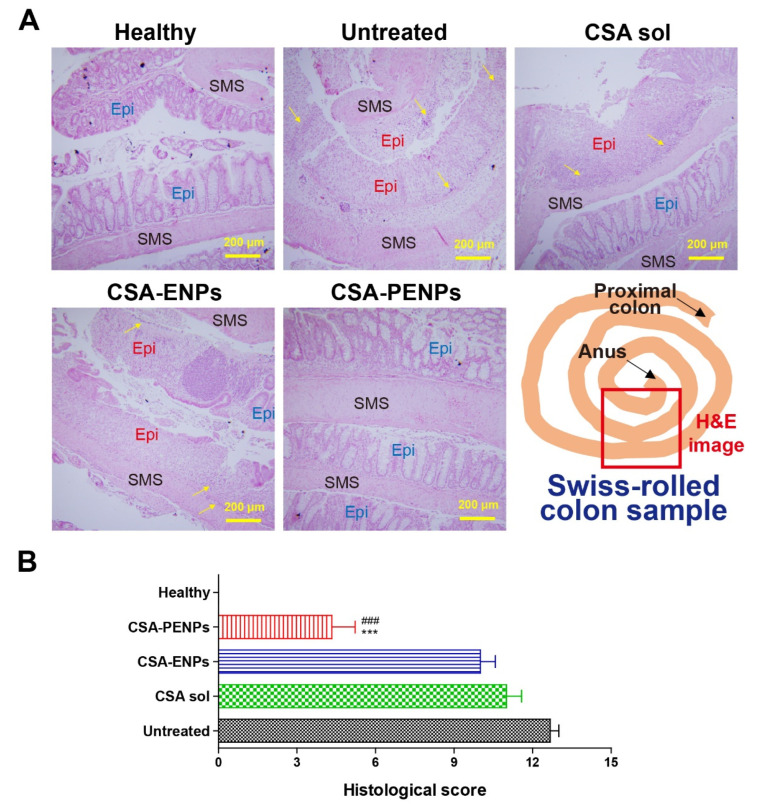
Histological assessments. (**A**) Representative H&E images of the colon treated with or without the CSA formulations. Black “SMS” indicates serosa, muscularis externa, and submucosa. Red and blue “Epi” indicate damaged and normal epithelium, respectively. Yellow arrow indicates infiltrated immune cells. Scale bar represents 200 µm. (**B**) Histological scores. *** denotes *p* < 0.001 compared to the untreated colitis group. ^###^ denotes *p* < 0.001 compared to the CSA-ENP-treated group. Data are expressed as mean ± standard deviation (n = 3).

After macroscopic assessments, the enhanced anti-inflammatory effects of CSA-PENPs were examined via histological observation. By using the Swiss-roll method to prepare samples for H&E staining, multiple points of each colon could be observed; however, when the traditional method was employed, only one point of the colon could be observed [41]. As shown in Figure 6A, in the H&E images of the untreated colitis group, the broad area of the outermost lining (mucosa, colon epithelium) was severely damaged, but displayed normal morphology, including a well-differentiated epithelium structure, in the healthy images. In the CSA solution and CSA-ENP-treated groups, pathological histological morphologies, such as epithelial depletion, absence of goblet cells in the mucosa, and massive immune cell infiltration resulting from the presence of inflammation in the colon, were observed. In contrast, in the H&E image from the CSA-PENP-treated group, the signs of inflammation were remarkably decreased, resulting in the restoration of the colon epithelium and reduced immune cell infiltration. For semi-quantitative analysis of the histological assessments, histological scores were measured from the H&E images (Figure 6B). Owing to the extensive tissue damage over a large area, the untreated group had the highest histological score among the experimental groups. The CSA solution and CSA-ENP-treated groups also had similar histological scores, which slightly decreased owing to the presence of partially recovered areas. In the CSA-PENP-treated group, histological score was significantly decreased relative to that in the CSA-ENP-treated and untreated colitis groups (*p* < 0.001) owing to the enhanced tissue recovery. However, as the tissues were not completely restored and the damaged portions still existed, the histological score of the CSA-PENPs was approximately 3.5, which is higher than that of the healthy group. Collectively, based on the histological assessments, CSA-PENPs exhibited potent re-epithelialization effects by alleviating inflammatory reactions in the colon. As CSA-PENPs could deliver a sufficient amount of CSA to the target tissues, enhanced anti-inflammatory effects could be achieved.

## 4. Conclusions

In this study, biosynthesized PHB-based pH-responsive CSA-loaded nanoparticles were developed for the colitis-targeted delivery of CSA. PHB was biosynthesized using *C. necator*, and its successful preparation was confirmed using ^1^H-NMR spectroscopy. After PHB characterization, CSA-loaded CSA-PENPs (6.28 ± 0.20%) were fabricated with a mean particle size of 311.4 ± 9.1 nm and zeta potential of −18.4 ± 0.06 mV. Owing to the pH responsive and sustained drug release effects provided by EFS and PHB, CSA release from CSA-PENPs in the simulated pH conditions of the stomach and small intestine was successfully prevented. In addition, although CSA-ENPs exhibited premature burst release after exposure to the proximal colon environment, CSA-PENPs exhibited prolonged CSA release for more than 24 h, resulting in sufficient CSA delivery to the inflamed tissues located in the distal colon. Finally, in the DSS-induced colitis mouse model, the CSA-PENPs group, displayed potent therapeutic effects compared with the untreated colitis and CSA-ENP-treated groups owing to the enhanced targeted delivery of CSA to the inflamed tissues. These results suggest that CSA-PENPs could be a promising drug-delivery system for the treatment of UC.

## Figures and Tables

**Table 1:** Characterization of CSA formulations.

	Size (nm)	PDI	Zeta Potential (mv)	CSA Loading (%)
**CSA-ENPs**	290.0 ± 26.3	0.119 ± 0.050	−19.4 ± 0.4	7.36 ± 0.23
**CSA-PENPs**	311.4 ± 9.1	0.136 ± 0.010	−18.4 ± 0.06	6.28 ± 0.20

Results are expressed as mean ± standard deviation (n = 3).

## Data Availability

All data are presented in the article.
